# Evaluation of Resistance of Banana Genotypes with AAB Genome to Fusarium Wilt Tropical Race 4 in China

**DOI:** 10.3390/jof8121274

**Published:** 2022-12-05

**Authors:** Ni Zhan, Mengyu Kuang, Weidi He, Guiming Deng, Siwen Liu, Chunyu Li, Nicolas Roux, Miguel Dita, Ganjun Yi, Ou Sheng

**Affiliations:** 1 Institute of Fruit Tree Research, Guangdong Academy of Agricultural Sciences, Key Laboratory of South Subtropical Fruit Biology and Genetic Resource Utilization, Ministry of Agriculture and Rural Affairs, Guangdong Provincial Key Laboratory of Tropical and Subtropical Fruit Tree Research, Guangzhou 510640, China; 2 College of Life Science, Langfang Normal University, Langfang 065000, China; 3 Alliance Bioversity International-CIAT, Parc Scientifique Agropolis II, CEDEX 5, 34397 Montpellier, France; 4 Bioversity International, The Americas Hub, Cali 763537, Colombia

**Keywords:** Fusarium wilt, tropical race 4, evaluation, Plantain, Iholena, Maia Maoli/Popoulu, Silk, Pisang Raja, Mysore

## Abstract

Banana cultivars with the AAB genome group comprise diverse subgroups, such as Plantain, Silk, Iholena, and Pisang Raja, among others, which play an important role in food security in many developing countries. Some of these cultivars are susceptible to *Fusarium oxysporum* f. sp. *cubense* tropical race 4 (*Foc* TR4), the most destructive pathogen threatening banana production worldwide, and some of them are still largely unknown. We evaluated the resistance of 37 banana genotypes, including Plantain, Silk, Iholena, Maia Maoli/Popoulu, Pisang Raja, Pome, and Mysore, to *Foc* TR4 under both greenhouse and field conditions. Genotypes from the Silk and Iholena subgroups were highly susceptible to *Foc* TR4. Pome and Mysore showed resistance and intermediate resistance, respectively. However, Pisang Raja ranged from susceptible to intermediate resistance. One cultivar from the Maia Maoli/Popoulu subgroup was highly susceptible, while the other displayed significant resistance. Most Plantain cultivars exhibited high resistance to *Foc* TR4, except two French types of cultivar, ‘Uganda Plantain’ and ‘Njombe N°2’, which were susceptible. The susceptibility to *Foc* TR4 of some of the AAB genotypes evaluated, especially Plantain and other cooking bananas, indicates that growers dependent on these varieties need to be included as part of the prevention and integrated *Foc* TR4 management strategies, as these genotypes play a crucial role in food security and livelihoods.

## 1. Introduction

**Figure 1:**
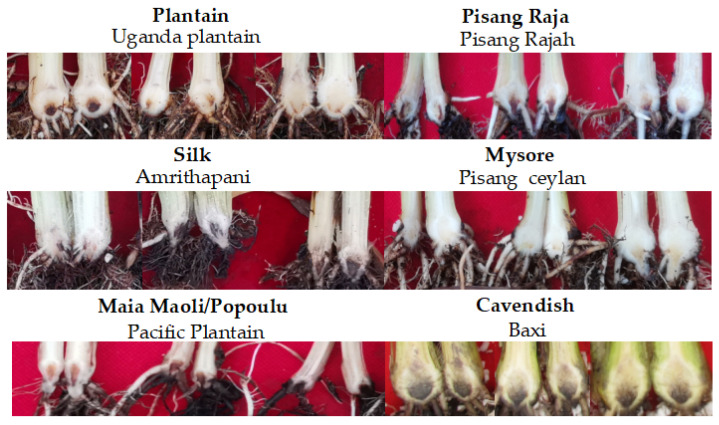
Longitudinal rhizome symptoms of cultivars grown in the greenhouse 35 days after inoculation with *Foc* TR4. Baxi was the susceptible control.

Bananas (*Musa* spp. L.) are one of the world’s most important cash crop grown on large plantations for export, and an essential staple food for many developing countries. Most cultivated bananas are seedless triploid varieties (2n = 3× = 33) derived from intra- or inter-specific hybridization between the two species *M*. *acuminata* (A genome) and *M. balbisiana* (B genome), resulting in the genome groups AAA, AAB and ABB [1]. The major cultivars are assigned to clusters of subgroups, which are characterized by genotypes that share similar traits of agronomic and fruit quality, such as Cavendish (AAA), East African Highland bananas (EAHB, AAA), and Plantain (AAB) [2].

Fusarium wilt of banana (FWB), caused by the soil-borne fungus *Fusarium oxysporum* f. sp. *cubense* (*Foc*), has been considered one of the most devastating diseases in agricultural history [3]. The pathogen can be disseminated by plant material, soil, and water [4]. Once *Foc* is introduced into a banana field, it cannot be eradicated and survives for many decades (as it forms resistant structures called chlamydospores); moreover, losses of up to 100% may occur depending on the banana cultivar’s susceptibility [5]. Upon pathogen infection, FWB symptoms start to appear, first on older leaves as they turn yellow and wilt, and subsequently developing on the younger leaves until the death of the whole plant [6]. Internally, the rhizome of the infected plants becomes discolored, and necrosis of the xylem vessels in the pseudostem occurs [7,8]. It is genetically diverse and capable of infecting a broad range of banana varieties [9].

Based on its pathogenicity to a group of differential banana cultivars, *Foc* is classified into three races. *Foc* race 1 (R1) affects a range of cultivars, such as Gros Michel (AAA), Ducasse (ABB, Pisang Awak), ‘Sugar’ (AAB, Silk), and ‘Lady Finger’ (AAB, Pome) [10,11]. This race was particularly known as it wiped out the Gros Michel-based exports in the banana industry in the last century [12]. *Foc* race 2 (R2), which has a banana cultivar host range overlapping with R1, is recognized for its potential to infect the cultivars of the Bluggoe subgroup (ABB), as well as closely related cooking bananas [10,11,12]. Apart from infecting the Cavendish subgroup cultivars, *Foc* race 4 (R4) also forms an overlapping continuum of host range potential with *Foc* R1 and *Foc* R2. After its initial discovery, R4 infected the Cavendish plants only in subtropical climates or plants under stress. These *Foc* populations were called as *Foc* subtropical race 4 (SR4) [11,12]. However, the existence of this structure for *Foc* might be controversial. For example, it was reported that a race 1 strain (VCG 0126) causing disease in Gros Michel is phenotypically and genetically similar to R4 [13,14].

A different and highly aggressive strain of *Foc* R4 was identified in 1990 that affected Cavendish plantations under tropical conditions without any biotic or abiotic stresses. The strain, called tropical race 4 (TR4) can affect a broader range of banana cultivars, including those infected by R1 and R2 [12,15]. *Foc* TR4 has spread from Asia to Australia, the Middle East and Africa, and was also recently reported in Colombia and Peru in Latin America [16]. A risk-of-spread scale indicated that *Foc* TR4 could affect 1.65 million ha of banana plantations globally by 2040, on which approximately 36 million tons of bananas are produced, with an estimated value of over USD 10 billion [17]. *Foc* TR4 threatens the livelihoods and income of millions in poor rural communities that rely on bananas. Therefore, effective management strategies to reduce the impact of this disease are urgently needed. In spite of the value of cultural management tactics related to soil health for instance, most researchers agree that the use of resistant varieties is the most effective means to manage this disease [9].

It is well documented that *Foc* TR4 can cause severe epidemics in ‘Cavendish’ (AAA), ‘Gros Michel’ (AAA), ‘Silk’ (AAB) and ‘Pisang Awak’ (ABB) (ABB) [12]. It has also been reported that Plantains (AAB) and East African Highland Bananas (EAHB, AAA) are resistant to TR4 [18]. Authors showed that 14 cultivars from EAHB and Plantain were evaluated to determine resistance to *Foc* TR4; the results showed that, with the exception of ‘Ibwi’ (EAHB subgroup), all the African cultivars sustained relatively low levels of disease ranging from 0–5%, and ‘Ibwi’ developed FWB symptoms at an incidence level of 32% [19]. A total of 129 accessions of *Musa* germplasm were evaluated for *Foc* TR4 resistance; these included 29 from the AA group, two from the BB group, 39 from the AAA group, 7 from the AAB group belonging to Plantains, and 11 from the ABB group. Of these, ‘Pahang’ (AA), ‘Calcutta 4’ (AA) and *M. itinerans* exhibited the highest degree of resistance, with an index of disease of less than 10. Furthermore, 31 cultivars from the AA, AB, AAB, AAAB, and ABB groups and their wild relatives were identified as resistant cultivars [18].

A banana cultivar-screening trial took place in the Northern Territory of Australia, which examined the responses of 24 banana cultivars to the soil-borne fungus, including three AA groups, nine AAA groups, two ABB groups, two AAB groups, and another tetraploid group. Several cultivars displayed considerable resistance to *Foc* TR4, including some FHIA parental lines and hybrids. The ‘Cavendish’ (AAA) somaclonal selections ‘GCTCV 215’ and ‘GCTCV 247’ from TBRI and an Indonesian selection, ‘CJ19’, showed very little to no plant death due to the disease [20].

Existing AAB cultivars, such as those from the Plantain and Iholena subgroups, are poorly evaluated and underrepresented for their resistance to *Foc* TR4. Hence, it is of critical importance to screen banana cultivars so that the novel *Foc* TR4-resistant cultivars can be identified and developed [21]. The screening of banana germplasms for *Foc* TR4 resistance can be achieved through pot or field trials. Pot trials enable the evaluation of a large number of different banana cultivars in a controlled environment, as well as screening to detect the resistant ones in the short run [22]. Field screening trials offer the opportunity to screen plants in sites affected by *Foc* TR4, where the resistant cultivars would be planted, to provide data on agronomical and yield performance as well as useful insights into the market’s potential receptivity [23]. In the present research, pot and field trials were conducted to assess the *Foc* TR4 reaction of 37 banana AAB cultivars from the Plantain, Iholena, Maia Maoli/Popoulu, Silk, Pisang Raja, Pome, and Mysore subgroups.

## 2. Materials and Methods

### 2.1. Plant Materials

The germplasms of 37 banana cultivars were evaluated in this study (Table 1), and were provided by the International *Musa* germplasm Transit Center (ITC), the Centre Africain de Recherche sur Bananiers et Plantains (CARBAP), and the Indonesian Tropical Fruit Research Institute (ITFRI). Previously described methods were adopted for performing rapid tissue culture propagation and plantlet regeneration [24].

### 2.2. Evaluation under Greenhouse Conditions

The strain Vegetative Compatibility Group (VCG 01213/16, Agriculture Culture Collection China, ACCC 37997) was used for inoculation under greenhouse conditions. It was originally collected from the Cavendish (AAA) cultivar ‘Baxi’ in Guangdong province, China. Previous work confirmed this strain as representing isolates of *Foc* TR4 [25,26], and it was used in our former germplasm screening [20]. The strain was initially cultured on potato dextrose agar (PDA) medium for 5 days, and then, the mycelium was transferred to potato dextrose broth (PDB) to grow for another 5 days at 28 °C with a shaking speed of 180 rpm. The final concentration of spore suspension was adjusted to 10^6^ spores/mL [27].

Four-month-old plantlets from each genotype were inoculated by immersing the roots in the *Foc* TR4 spore suspension for 30 min, then transferred to sterile perlite. Three replications (six plantlets for each replication) were used for each genotype. Plantlets of the Cavendish (AAA) cultivar ‘Baxi’ were used as a susceptible control. After inoculation, plantlets were maintained in the greenhouse at 25–28 °C, with 70–80% relative humidity. Disease evaluation was performed at 35 days after inoculation when typical FWB symptoms were observed in the susceptible control ‘Baxi’. The disease severity was assessed via the Rhizome Discoloration Index (RDI) according to the rhizome discoloration (internal symptom) ratio (rating scale) as follows: 1: the absence of internal symptoms; 2: the occurrence of several internal spots; 3–5: <1/3, 1/3–2/3, and >1/3 areas discolored; 6: the whole inner rhizome discolored. Additionally, the genotypes were classified as ‘susceptible’ (S, RDI > ‘3’), ‘intermediate’ (I, RDI = ‘2’–‘3’) or ‘resistant’ (R, RDI < ‘2’) [28].

### 2.3. Evaluation under Field Conditions

Between November 2019 and January 2022, field tests were conducted to evaluate the genotypes for their resistance to *Foc* TR4 in naturally infested soils located in Dongguan, Guangdong province (N23°02′17.25″, E113°40′52.87″). This site for field testing was the same place in which we previously carried out germplasm screening of bananas for resistance to *Foc* TR4 [18]. The Cavendish (AAA) cultivar ‘Baxi’, used as susceptible control, was previously planted in these plots with >80% of FWB TR4 incidence. The experimental plot was arranged as a randomized complete block design with three replications (blocks), and 10–15 plants within each block for each genotype. Each plant was cultivated according to local commercial growing standards for two cropping cycles, with inter-row spacing of 3.5 m and inter-plant spacing of 2.0 m [29]. No chemicals were applied to control pests and diseases. Disease evaluation was conducted when external symptoms of FWB, such as leaf yellowing on the oldest leaves and occasional pseudostem splitting appeared on the susceptible control ‘Baxi’. Once plants were dead or harvested, internal symptom on the rhizome were evaluated. The incidence of disease (ID) was calculated when plants were dead or harvested (2 years after planting) as follows: ID (%) = [diseased plants/total plants] × 100. According to the ID values, the following categories of disease reaction were established: 0% ≤ ID ≤ 20%: highly resistant (HR); 20% < ID ≤ 40%: resistant (R); 40% < ID ≤ 60%: intermediate (I); 60% < ID ≤ 80%: susceptible (S); and 80% ≤ ID: very susceptible (VS) [18].

### 2.4. Molecular Characterization of Foc TR4

To verify the presence or identity of *Foc* TR4, rhizome tissues (10 cm × 10 cm) were sampled from the discolored parts of the plants if they showed typical Fusarium wilt symptoms, or randomly sampled if no symptoms were observed. The samples were analyzed via PCR using *Foc* TR4 specific primers [27]. At least one sample per replicate was collected from each cultivar. All the samples were surface-sterilized with 70% ethanol for 5 min, washed with sterile distilled water, and allowed to dry on the sterile filter paper. Thereafter, they were cut into segments of 2 mm^2^ and added to tubes containing 200 µL cell lysate; then, they were ground, boiled for 5 min, and centrifuged at 12,000 rpm for 2 min. PCR was performed with 2 µL of the sample as the template for PCR amplification using the Mix MF848 kit (Mei5 Biotechnology Co., Beijing, China) under the following PCR conditions: 3 min at 95 °C, 30 s at 94 °C, 40 s at 56 °C, 1 min at 72 °C for 35 cycles, and 5 min at 72 °C. In the control group, we used genomic DNA (gDNA) extracted from *Foc* TR4 (VCG 01213/16, ACCC 37997) but not from banana plants. *Foc* TR4-specific primers were used to detect *Foc* TR4 [27].

### 2.5. Data Collection and Statistical Analysis

ANOVA was adopted using SPSS 19.0 statistics for comparing different banana cultivars with varying levels of resistance to Fusarium wilt, assessed under greenhouse as well as field conditions, using resistance parameters such as ID and RDI. The Fisher’s least significant difference (LSD) test was applied for multiple comparisons of variables at 0.05 (*p* < 0.05). 

## 3. Results

### 3.1. Evaluation under Greenhouse Conditions

**Figure 2:**
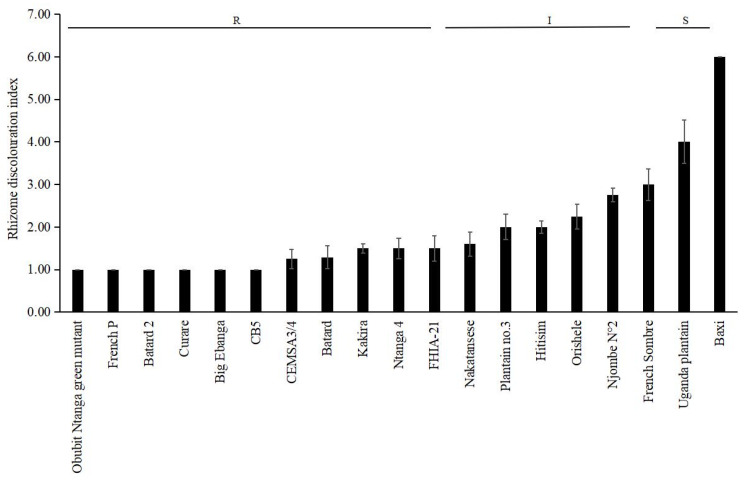
Rhizome discoloration index (RDI) of Plantain cultivars grown 35 days after inoculation with *Fusarium oxysporum* f. sp. *cubense* tropical race 4 in the greenhouse. Cavendish cultivar ‘Baxi’ was the susceptible control. The data are presented as mean ± SD (standard deviation) of three replicates. R: resistant; I: intermediate; S: susceptible.

The susceptible control, the Cavendish (AAA) cultivar ‘Baxi’, developed internal rhizome symptoms of FWB TR4 at 35 days after inoculation, with an RDI value of 6. Plantain cultivars including ‘Uganda Plantain’, ‘French Sombre’, and ‘Njombe N°2’ cultivars were infected by *Foc* TR4, with RDI values of 4, 3, and 2.75, respectively (Figure 1 and Figure 2). Six Plantain genotypes showed RDI values of one and eight Plantain cultivars values ranging from 1 to 2.25 (Figure 2).

**Figure 3:**
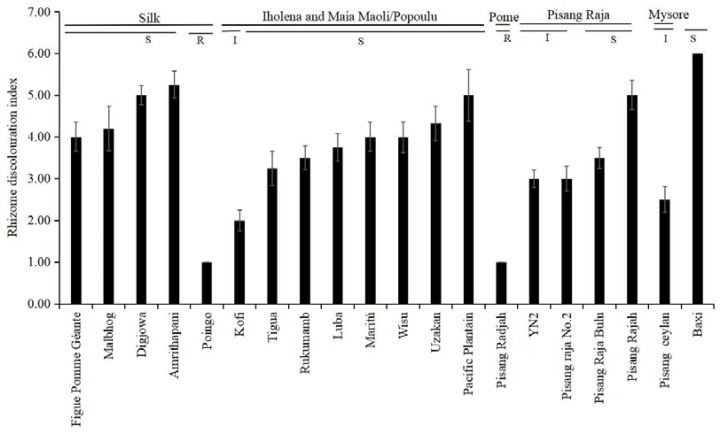
Rhizome discoloration index of the other cultivars belonging to the genome group AAB, grown for 35 days in the greenhouse. Cavendish cultivar ‘Baxi’ was the susceptible control. The data are presented as mean ± SD (standard deviation) of three replicates. R: resistant; I: intermediate; S: susceptible.

All the genotypes from the Silk subgroup were severely infected by *Foc* TR4, showing RDI values higher than 4, with no difference compared to the susceptible control (Figure 1 and Figure 3). Most genotypes of the Iholena and Maia Maoli/Popoulu subgroup were infected by *Foc* TR4. ‘Luba’, ‘Maritú’, ‘Wisu’, ‘Uzakan’, and ‘Pacific Plantain’ showed susceptibility (Figure 1 and Figure 3). No disease symptoms were observed in ‘Poingo’. There were four cultivars in the Pisang Raja subgroup, with RDI values ranging from 3 to 5. ‘Pisang Rajah’ had an RDI value of 5 and ‘Pisang Radjah’ had an RDI value of 1, and no disease symptoms were observed in ‘Pisang Radjah’, which belonged to the Pome subgroup (Figure 1 and Figure 3). ‘Pisang Ceylan’ belonging to the Mysore subgroup had an RDI value of 2 (Figure 3).

As shown in Table 2, the susceptible Cavendish control (‘Baxi’) exhibited a resistance rating of susceptible. Likewise, ‘Uganda Plantain’ was susceptible, while among the other Plantain cultivars, six were classified as resistant, and the other five were assigned as intermediate. All of the four Silk banana cultivars were classified as susceptible. Moreover, ‘Poingo’ had a rating of resistant, whereas ‘Kofi’ was classified as intermediate, and the other seven Iholena cultivars were rated as susceptible. Similarly, ‘Pisang Radjah’ was given susceptible rating. ‘YN2’ and ‘Pisang Raja No.2’ displayed intermediate ratings, and ‘Pisang Raja Bulu’ and ‘Pisang Rajah’ had ratings of susceptible. A rating of intermediate was obtained for ‘Pisang Ceylan’ belonging to the Mysore subgroup (Table 2).

### 3.2. Evaluation under Field Conditions

**Figure 4:**
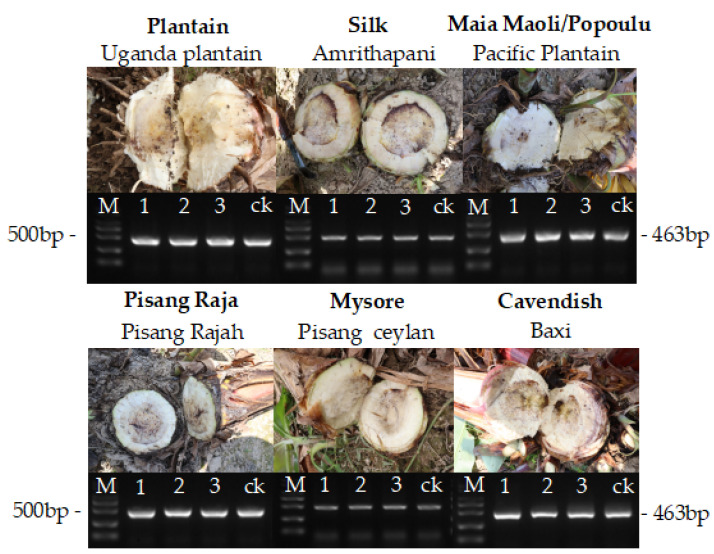
Symptoms of Fusarium wilt tropical race 4 (TR4) in rhizomes of banana genotypes under field conditions and amplification of PCR products confirming the presence of *Fusarium oxysporum* f. sp. *cubense* TR4 via PCR analyses (M: marker. 1, 2, 3: replicates per genotype; ck: reference *Foc* TR4 strain: VCG 01213/16, ACCC 37997). ‘Baxi’ was the susceptible control.

Internal symptoms typical of FWB were first observed in November 2019 (Figure 4). Infection caused by *Foc* TR4 was confirmed using PCR analyses (Figure 4). To facilitate the comparison and further discussions, disease reaction will be presented by subgroups referring to the susceptible control ‘Baxi’.

**Figure 5:**
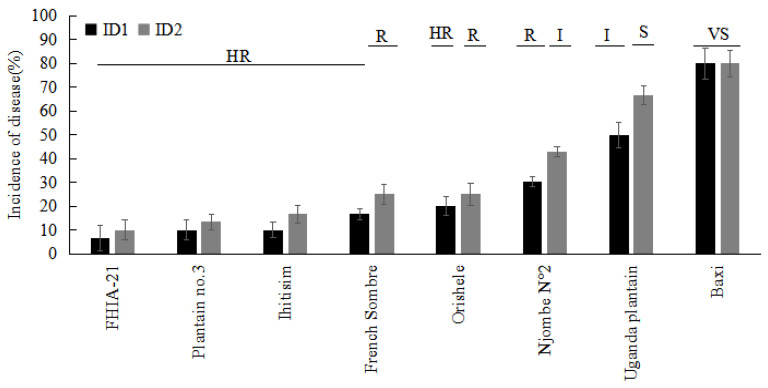
Incidence of Fusarium wilt Tropical race 4 on Plantain genotypes under field conditions after two cropping cycles. Cavendish cultivar ‘Baxi’ was the susceptible control. The data are presented as mean ± SD (standard deviation) of three replicates. ID1: plant crop; ID2: first ratoon; HR: highly resistant; R: resistant; I: intermediate; S: susceptible; VS: very susceptible.

Of the 19 Plantain genotypes evaluated, only seven showed symptoms of FWB (Figure 5). ‘Uganda Plantain’ and ‘Njombe N°2’ showed the highest disease intensity, with ID values of 50% and 30.33%, respectively (Figure 5). The other Plantain genotypes affected by *Foc* TR4 were FHIA-21, Plantain no.3, Ihitisim, French Sombre, and Orishele, with ID values ranging from 10% to 20 % (Figure 5). The remaining genotypes (‘Obubit Ntanga green mutant’, ‘Kakira’, ‘French P’, ‘Nakatansese’, ‘Ntanga 4’, ‘Batard’, ‘Batard 2’, ‘Curare’, ‘Big Ebanga’, ‘CB5’, and ‘CEMSA3/4’) did not exhibit any internal symptoms of FWB over the two cropping cycles (Figure 5). 

**Figure 6:**
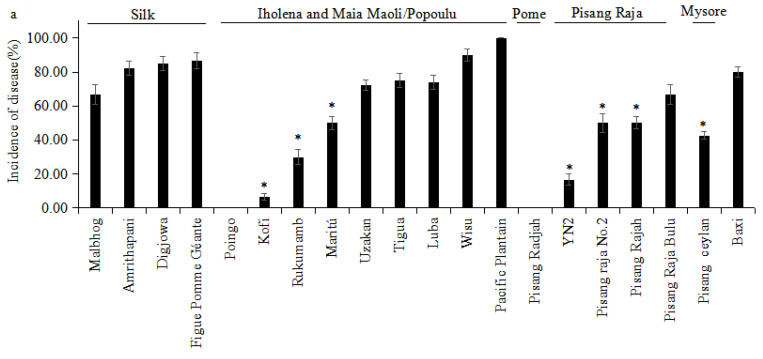

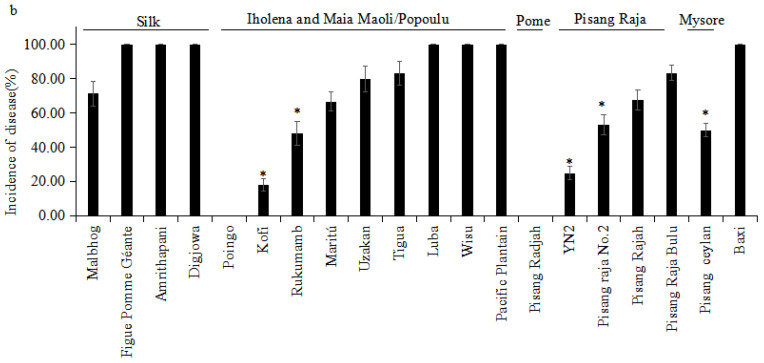
Incidence of Fusarium wilt Tropical race 4 in banana genotypes in the (**a**) plant crop and (**b**) first ratoon; Cavendish cultivar ‘Baxi’ was the susceptible control. The data are presented as mean ± SD (standard deviation) of three replicates. Cultivars marked with an asterisk indicate that the mean is significantly different to the susceptible control, Cavendish cultivar ‘Baxi’ (*p* < 0.05).

All the Silk-type bananas were susceptible to *Foc* TR4, showing ID values even higher than ‘Baxi’ (Figure 6). Three genotypes (‘Amrithapani’, ‘Digjowa’, and ‘Figue Pomme Géante’) presented 100% disease incidence after the second cropping cycle, which also evidenced the high *Foc* TR4 inoculum pressure in the experimental plots (Figure 6). Most of genotypes from the Iholena subgroup and one from the Maia Maoli/Popoulu subgroup were highly affected by *Foc* TR4, with the highest ID values occurring in ‘Maritú’, ‘Uzakan’, ‘Digjowa’, ‘Luba’, ‘Wisu’, and ‘Pacific Plantain’ (Figure 6). ‘Kofi’ and ‘Rukumamb’ presented ID values ranging from 6.67% to 48.25%. Interestingly, ‘Poingo’ from the Maia Maoli/Popoulu subgroup did not develop any internal symptoms of the disease (Figure 6). Genotypes from the Pisang Raja subgroup also presented variation regarding *Foc* TR4 resistance (Figure 6). ‘Pisang Raja Bulu’, ‘Pisang Raja No.2’, and ‘Pisang Rajah’ were considered susceptible. ‘YN2’ presented only a 15% ID and the Pome cultivar ‘Pisang Radjah’ did not show any FWB symptoms (Figure 6). These were considered intermediate and highly resistant, respectively. The intermediate genotype ‘Pisang Ceylan’ from the Mysore subgroup showed an ID value of around 40% (Figure 6).

The Cavendish cultivar ‘Baxi’ (susceptible control) was rated as very susceptible (VS) during both cropping cycles. In the Plantain subgroup, ‘Obubit Ntanga green mutant’, ‘French P’, ‘Batard 2’, ‘Curare’, ‘Big Ebanga’, ‘CB5’, ‘CEMSA3/4’, ‘Batard’, ‘Kakira’, ‘Ntanga 4’, ‘FHIA-21’, ‘Nakatansese’, ‘Plantain no.3’, and ‘Ihitisim’ were ranked as highly resistant in both crop cycles. ‘Orishele’ was highly resistant in the crop cycle but resistant in the first ratoon crop cycle. ‘Njombe N°2’ was observed to be resistant in the crop cycle but intermediate in the first ratoon crop cycle. ‘French Sombre’ was designated as highly resistant during the crop cycle, whereas during the first ratoon crop cycle, it was resistant. Moreover, ‘Uganda Plantain’ was ranked as resistant during the first cropping cycle, but susceptible during the second (ratoon) cropping cycle (Table 2).

In the Iholena and Maia Maoli/Popoulu subgroups, ‘Poingo’ and ‘Kofi’ were designated as highly resistant, and ‘Wisu’ and ‘Pacific Plantain’ were rated as very susceptible during both crop cycles. ‘Tigua’ was noticed to be susceptible during the plant crop cycle and very susceptible during the initial ratoon crop cycle. Additionally, ‘Rukumamb’ displayed resistance to *Foc* TR4 during the plant crop cycle but intermediate levels of resistance in the first ratoon crop cycle. Susceptibility was noticed in ‘Luba’ during the plant crop cycle; however, during the initial ratoon crop cycle, the cultivar was very susceptible. ‘Maritú’ showed intermediate-level resistance during the crop cycle but susceptibility during the initial ratoon crop cycle. Furthermore, ‘Uzakan’ was rated as susceptible during the crop cycle but quite susceptible during another crop cycle (Table 2). ‘Figue Pomme Géante’, ‘Digjowa’, and ‘Amrithapani’ were designated as very susceptible, and ‘Malbhog’, which belonged to Silk subgroup, was shown to be susceptible during both crop cycles (Table 2). For the Pisang Raja subgroup, ‘YN2’ developed high-level resistance in the crop cycle, whereas, throughout the first ratoon crop cycle, it was resistant. The evaluated ‘Pisang Raja Bulu’ was found to be susceptible during the crop cycle but very susceptible during the initial ratoon cycle. ‘Pisang Rajah’ demonstrated intermediate-level resistance and susceptibility during the crop cycle and the initial ratoon crop cycle, respectively. The Pome cultivar ‘Pisang Radjah’ displayed high resistance, and ‘Pisang Ceylan’ belonging to Mysore subgroup showed intermediate resistance in the plant crop and ratoon crop cycles, respectively (Table 2).

## 4. Discussion

The use of resistant varieties could be the most efficient measure to manage *Foc* TR4, which is currently recognized as the most devastating disease in bananas [30,31,32]. In this work, we assessed the resistance level of 37 banana cultivars belonging to the AAB genome against *Foc* TR4 under both greenhouse and field conditions. The resistance of some cultivars evaluated in the field trial was lower than that obtained in the greenhouse, which might be due to the inoculum concentration and uncontrolled soil characteristics in the field [28].

Plantains, a very important subgroup referred to as cooking bananas (the AAB genome), are of major importance in the diets of numerous populations of Africa, Latin America, and the Caribbean [33]. Our previous results grouped some Plantain cultivars as highly resistant (HR) or resistant (R) [18]. However, in the current results, two and five cultivars were found to be intermediate from field and greenhouse evaluation, respectively. This indicates that some Plantain genotypes, despite displaying a resistant phenotype, are infected by *Foc* TR4 and, consequently, could disseminate the pathogen through the planting material, for instance. The ‘Uganda Plantain’ genotype was susceptible, ruling out the hypothesis that all Plantain genotypes display some level of resistance to *Foc* TR4. Altogether, our results also suggest that French types of Plantain could be more susceptible to *Foc* TR4, though more studies are still necessary to address this hypothesis.

Banana genotypes belonging to the Iholena subgroup are distinguished by the orange color of their pulp fruits, which is indicative of high pro-vitamin A carotenoid content [34]. Previous result showed that this subgroup is susceptible to *Foc* R1, but its reaction to *Foc* TR 4 is almost unknown [7]. In the present results, only one cultivar from this subgroup was ranked as resistant to *Foc* TR4, and the other six cultivars were susceptible (Table 2). Interestingly, the two cultivars from the Maia Maoli/Popoulu subgroup, ‘Pacific Plantain’ and ‘Poingo’, exhibited different resistance levels to *Foc* TR4, which might be associated with diverse resistance genes within the genome.

The Silk subgroup contains genotypes bearing sweet acidic fruits with an apple-like flavor and are well known for their susceptibility to *Foc* R1 [35]. Indeed, our results showed that all Silk genotypes evaluated were also susceptible to *Foc* TR4. Viljoen et al. (2017) [28] also found similar results, indicating that Silk genotypes can be used as susceptible references in phenotyping assays for *Foc* TR4 resistance. Pisang Raja is one of the most economically important local banana cultivars, in Indonesia, particularly in Java [36]. From our results, only one cultivar from this subgroup was found to be resistant, whereas the others were shown to be intermediate or susceptible (Table 2). Pome banana is popularly consumed in India and Australia, and particularly in Brazil, where this subgroup is known as Prata [2]. Mysore banana is widely used in countries such as India, due to its functional and nutraceutical properties, along with great swelling ability to elaborate jellies and meat-based products [37]. In this paper, the Pome cultivar ‘Pisang Radjah’ and the Mysore cultivar ‘Pisang Ceylan’ showed resistance and intermediate resistance to *Foc* TR4, respectively. However, it was reported that the Pome cultivar ‘Lady Finger’ was susceptible to *Foc* race 1 and subtropical race 4 in the field [38], whereas it was severely infected by *Foc* TR4 in a shade house pot trial [22].

It is interesting that the screening result of a total of 258 genotypes against *Foc* race 1 (VCG 0124) revealed that different reactions (from immune to susceptible) existed in Pome and Mysore subgroups [39]. These results indicate that more cultivars from these two subgroups should be included for further evaluation on the reaction to *Foc* TR4.

In the field evaluation, some genotypes (Dwarf Nathan, SH-3436, and FHIA-03) showed increased susceptibility throughout their cropping cycles, which was also reported by Mintoff et al. (2021) [20] and Ndayihanzamaso et al. (2020) [40]. The increased inoculum density of *Foc* TR4 could be related to this fact, as infected plants were not eradicated. Therefore, the secondary inoculum generated by infected plants, mainly from Baxi (susceptible control), and by susceptible genotypes increased after each cropping cycle.

## 5. Conclusions

In summary, in this work we assessed the resistance level of 37 banana cultivars against *Foc* TR4, identifying sources for disease resistance which might support plant breeding. The fact that Plantains can be infected by *Foc* TR4 is particularly relevant to food security in Africa, Latin America, and the Caribbean, where these genotypes are staple foods, but also provide a major source of income. Therefore, more efforts are needed to evaluate resistance in global Plantain germplasm banks to *Foc* TR4. In addition, many other varieties play important roles on food security in many countries around the world where *Foc* TR4 is not yet present. Therefore, information about the behaviors of these genotypes regarding *Foc* TR4 resistance might support National Plant Protection Organizations through risk analyses and phytosanitary policies.

## Figures and Tables

**Table 1:** Banana cultivars assessed for resistance to *Fusarium oxysporum* f. sp *cubense* tropical race 4.

Cultivar	ITC Code	Genome	Subgroup/Type
Obubit Ntanga green mutant	ITC0519	AAB	Plantain/French
Kakira	NA	AAB	Plantain/French
Uganda Plantain	NA	AAB	Plantain/French
French Sombre	ITC1668	AAB	Plantain/French
French P	NA	AAB	Plantain/French
Nakatansese	NA	AAB	Plantain/French
Ntanga 4	ITC0226	AAB	Plantain/French
Njombe N°2	ITC1124	AAB	Plantain/French
Batard	NA	AAB	Plantain/French Horn
Batard 2	NA	AAB	Plantain/French Horn
Plantain no.3	ITC0498	AAB	Plantain/French Horn
Orishele	ITC1325	AAB	Plantain/False Horn
Curare	ITC1165	AAB	Plantain/False Horn
Big Ebanga	NA	AAB	Plantain/False Horn
CB5	NA	AAB	Plantain/False Horn
CEMSA3/4	NA	AAB	Plantain/False Horn
Ihitisim	ITC0121	AAB	Plantain/Horn
FHIA-21	NA	AAAB	Hybrid/Plantain
Figue Pomme Géante	ITC0769	AAB	Silk
Amrithapani	ITC1612	AAB	Silk
Malbhog	ITC1631	AAB	Silk
Digjowa	ITC1633	AAB	Silk
Maritú	ITC0639	AAB	Iholena
Luba	ITC0802	AAB	Iholena
Wisu	ITC0880	AAB	Iholena
Kofi	ITC0912	AAB	Iholena
Tigua	ITC0875	AAB	Iholena
Uzakan	ITC0825	AAB	Iholena
Rukumamb	ITC0831	AAB	Iholena
Pacific Plantain	ITC0210	AAB	Maia Maoli/Popoulu
Poingo	ITC1327	AAB	Maia Maoli/Popoulu
Pisang Rajah	ITC0587	AAB	Pisang Raja
Pisang Raja Bulu	ITC0843	AAB	Pisang Raja
YN2	NA	AAB	Pisang Raja
Pisang Raja No.2	NA	AAB	Pisang Raja
Pisang Ceylan	ITC1441	AAB	Mysore
Pisang Radjah	ITC0243	AAB	Pome
Baxi	NA	AAA	Cavendish

NA: Not available.

**Table 2:** Reaction to Fusarium wilt tropical race 4 of banana genotypes under greenhouse and field conditions.

Cultivar	Subgroup/Type	Greenhouse	Plant Crop	First Ratoon
		RDI—Rating	ID (%)—Rating	ID (%)—Rating
Baxi	Cavendish	6.00—S	80.00—VS	80.00—VS
Big Ebanga	Plantain/False Horn	1.00—R	0.00—HR	0.00—HR
CB5	Plantain/False Horn	1.00—R	0.00—HR	0.00—HR
CEMSA3/4	Plantain/False Horn	1.25—R	0.00—HR	0.00—HR
Curare	Plantain/False Horn	1.00—R	0.00—HR	0.00—HR
Orishele	Plantain/False Horn	2.25—I	20.00—HR	25.00—R
Batard	Plantain/ French Horn	1.29—R	0.00—HR	0.00—HR
Batard 2	Plantain/ French Horn	1.00—R	0.00—HR	0.00—HR
Plantain no.3	Plantain/ French Horn	2.00—I	10.00—HR	13.33—HR
French P	Plantain/French	1.00—R	0.00—HR	0.00—HR
French Sombre	Plantain/French	3.00—I	16.67—HR	25.00—R
Kakira	Plantain/French	1.50—R	0.00—HR	0.00—HR
Nakatansese	Plantain/French	1.60—R	0.00—HR	0.00—HR
Njombe N°2	Plantain/French	2.75—I	30.33—R	42.86—I
Ntanga 4	Plantain/French	1.50—R	0.00—HR	0.00—HR
Obubit Ntanga green mutant	Plantain/French	1.00—R	0.00—HR	0.00—HR
Uganda Plantain	Plantain/French	4.00—S	50.00—I	66.67—S
Ihitisim	Plantain/Horn	2.00—I	10.00—HR	16.67—HR
FHIA-21	Hybrid/Plantain	1.50—R	6.67—HR	10.00—HR
Kofi	Iholena	2.00—I	6.67—HR	18.18—HR
Luba	Iholena	3.75—S	75.00—S	100.00—VS
Maritú	Iholena	4.00—S	50.00—I	66.67—S
Rukumamb	Iholena	3.50—S	30.00—R	48.25—I
Tigua	Iholena	3.25—S	75.00—S	83.33—VS
Uzakan	Iholena	4.33—S	72.25—S	80.00—VS
Wisu	Iholena	4.00—S	90.00—VS	100.00—VS
Pacific Plantain	Maia Maoli/Popoulu	5.00—S	100.00—VS	100.00—VS
Poingo	Maia Maoli/Popoulu	1.00—R	0.00—HR	0.00—HR
Pisang Raja Bulu	Pisang Raja	3.50—S	66.67—S	83.33—VS
Pisang Raja No.2	Pisang Raja	3.00—I	50.00—I	53.00—I
Pisang Rajah	Pisang Raja	5.00—S	50.00—I	67.66—S
YN2	Pisang Raja	3.00—I	16.67—HR	25.00—R
Pisang Raja	Pome	1.00—R	0.00—HR	0.00—HR
Pisang Ceylan	Mysore	2.50—I	42.75—I	50.00—I
Amrithapani	Silk	5.25—S	82.25—VS	100.00—VS
Digjowa	Silk	5.00—S	85.00—VS	100.00—VS
Figue Pomme Géante	Silk	4.00—S	86.67—VS	100.00—VS
Malbhog	Silk	4.20—S	66.67—S	71.43—S

HR: highly resistant (0% ≤ ID ≤ 20%); R: resistant (20% < ID ≤ 40%); I: intermediate (40% < ID ≤ 60%); S: susceptible (60% < ID ≤ 80%); VS: very susceptible (80% ≤ ID).

## Data Availability

Not applicable.
